# Interfacial Engineering of MgCo_2_O_4_@Ni(OH)_2_ Heterostructures for Efficient Alkaline Water Oxidation

**DOI:** 10.3390/molecules31203671

**Published:** 2026-10-14

**Authors:** Chaitany Jayprakash Raorane, Seong-Cheol Kim

**Affiliations:** School of Chemical Engineering, Yeungnam University, Gyeongsan 38541, Republic of Korea; sckim07@ynu.ac.kr

**Keywords:** oxygen evolution reaction, water splitting, electrodeposition, heterostructure, MgCo_2_O_4_@Ni(OH)_2_

## Abstract

Developing highly active, durable, and earth-abundant electrocatalysts is essential for advancing sustainable hydrogen production through electrochemical water oxidation. Herein, a hierarchical MgCo_2_O_4_@Ni(OH)_2_ heterostructure was constructed by electrodepositing Ni(OH)_2_ nanosheets onto MgCo_2_O_4_ nanorods and investigated as an electrocatalyst for alkaline water splitting. The intimate contact between the MgCo_2_O_4_ nanorod and Ni(OH)_2_ nanosheet creates a hierarchical architecture with abundant accessible surface sites and favorable pathways for electrolyte penetration and charge transport. Among the prepared electrodes, MCO@Ni(OH)_2_-2 exhibited the best OER performance, requiring an overpotential of 348 mV to achieve 10 mA cm^−2^ with a Tafel slope of 70.2 mV dec^−1^. The optimized electrode also showed a low charge-transfer resistance of 2.7 Ω, indicating favorable interfacial charge transport. Its hierarchical architecture provides a highly accessible electrochemical surface, shortened ion-diffusion pathways, and efficient electrolyte penetration, thereby promoting the adsorption and transformation of reaction intermediates. The enhanced performance is attributed to the optimized Ni(OH)_2_ nanosheet coverage, increased electrochemically active surface area, and synergistic interaction between the MgCo_2_O_4_ nanorod and Ni(OH)_2_. Furthermore, the electrode retained substantial electrocatalytic activity during prolonged cycling and continuous operation under alkaline conditions. These results demonstrate that engineering hierarchical oxide/hydroxide interfaces offers an effective approach for developing efficient transition-metal-based electrocatalysts for sustainable water splitting.

## 1. Introduction

The rapid depletion of fossil-fuel resources, coupled with escalating environmental concerns, has intensified the global demand for sustainable, environmentally benign, and scalable energy technologies [1,2,3,4]. The production of Hydrogen (H) with electrochemical water splitting provides an ideal way to create green and clean chemical energy carriers [5]. Electrochemical water splitting is composed of two half-reactions: a hydrogen evolution reaction (HER) at the cathode and an oxygen evolution reaction (OER) the anode, which both involve complicated catalytic reaction mechanisms [6,7]. Compared with the two-electron transfer mechanism for the HER, which requires a simpler catalytic active site [8], the OER involves four-electron-coupled proton transfer where high-energy intermediates (OH, O, *OOH) are produced sequentially and the stiff binding of the Oxygen-Oxygen (O=O) bond makes OER kinetics slow. A very high overpotential (η) (above thermodynamic potential, 1.23 V vs. RHE) is also required to overcome the kinetics hindrance; in fact, this OER process is the rate-limiting process and the main bottleneck of water splitting [8,9,10,11].

To overcome these kinetic hurdles, noble metal oxides, specifically ruthenium oxide (RuO_2_) and iridium oxide (IrO_2_), are traditionally deployed as state-of-the-art electrocatalysts due to their low overpotentials [7,12]. However, their high cost, scarcity, and susceptibility to dissolution under highly oxidizing conditions strictly limit their large-scale industrial viability. Consequently, substantial academic and industrial effort has transitioned toward developing earth-abundant, highly active, and durable transition metal-based alternatives [13]. Among these candidates, cobaltite-based transition metal oxides crystallizing in the spinel structure, such as magnesium cobaltite (MgCo_2_O_4_), have attracted considerable attention because of their spinel structure, redox-active transition-metal centers, structural stability, and potential for electrochemical applications. In alkaline environments, the transition between mixed-valence states (Co^3+^/Co^4+^) within the spinel matrix facilitates the formation of active surface oxyhydroxide (CoOOH) phases [14]. Nonetheless, pristine MgCo_2_O_4_ electrocatalysts typically suffer from poor intrinsic electronic conductivity and an insufficient density of accessible active surface sites, which curtails their practical catalytic throughput.

Surface engineering and the construction of heterostructured materials present an effective pathway to transcend these intrinsic performance limitations. Integrating a secondary active component, particularly nickel hydroxide [Ni(OH)_2_], has proven remarkably successful in creating synergistic electronic structures that optimize the adsorption energies of oxygenated intermediates [15,16]. Nickel hydroxide exists as a versatile structural platform that transforms dynamically during forward oxidative sweeps into highly conductive, amorphous β-NiOOH or mixed Ni/Co oxyhydroxides, which serve as highly active phases for oxygen evolution [17]. For example, a 2D Ni(OH)_2_ heterojunction with a sodium organic compound showed good OER activity [18]. Fe-Doped β-Ni(OH)_2_ nanosheets represent a major advancement in decreasing the overpotential for the oxygen evolution reaction [19]. The Ni(OH)_2_/GO nanocomposite shows an improved oxygen evolution reaction (OER) performance with a low overpotential [20]. Among various synthesis methodologies, electrochemical deposition offers a distinct, binder-free, and highly controllable technique for anchoring uniform Ni(OH)_2_ layers onto conductive or pre-formed catalyst matrices. This approach allows for the precise regulation of the local coordination environment, shortens ion-diffusion pathways, and maximizes the electrochemically active surface area (ECSA) without requiring structurally destructive, high-temperature calcination methods [21].

In this work, we present the strategic design and fabrication of a high-performance heterostructured electrocatalyst by electrodepositing a nanostructured Ni(OH)_2_ active layer onto a pre-synthesized MgCo_2_O_4_ matrix. The deliberate coupling of the electrodeposited Ni(OH)_2_ with the underlying MgCo_2_O_4_ spinel sets up an optimized heterointerface that drastically accelerates charge-transfer rates and balances intermediate adsorption during the OER. Comprehensive structural, chemical, and morphological analyses confirm the successful formation of the MgCo_2_O_4_@Ni(OH)_2_ heterostructure and the effective deposition of Ni(OH)_2_ nanosheets on the MgCo_2_O_4_ nanorod framework. When evaluated in an alkaline electrolyte (1.0 M KOH), the resulting MgCo_2_O_4_@Ni(OH)_2_ heterostructure displays an exceptionally lowered overpotential, reduced Tafel slope, and enhanced long-term durability compared to the pristine spinel matrix, offering a straightforward synthesis strategy for constructing MgCo_2_O_4_@Ni(OH)_2_ electrodes.

## 2. Experimental Section

### 2.1. Chemicals

Magnesium nitrate hexahydrate (Mg(NO_3_)_2_·6H_2_O, 98%), nickel nitrate hexahydrate (Ni(NO_3_)_2_·6H_2_O, 99.9%), cobalt nitrate hexahydrate (Co(NO_3_)_2_·6H_2_O, ≥99%), and urea (CO(NH_2_)_2_, 99.0%) were purchased from Sigma-Aldrich (St. Louis, MO, USA). Ammonium fluoride (NH_4_F) and ethanol (C_2_H_5_OH, 94.5%) were supplied by Duksan Chemicals (Ansan-si, Gyeonggi-do, Republic of Korea). Potassium hydroxide (KOH, >85%) was obtained from Daejung Chemicals & Metals (Siheung-si, Gyeonggi-do, Republic of Korea), while nickel foam (NF) was procured from NARA Cell-Tech Corporation (Geumcheon-gu, Seoul, Republic of Korea). All chemicals were of analytical grade and were used as received without further purification. Deionized (DI) water was employed throughout the experiments for precursor solution preparation, substrate cleaning, and other synthesis-related procedures.

### 2.2. Preparation of MgCo_2_O_4_ and MgCo_2_O_4_@Ni(OH)_2_ Series

**Figure 1:**
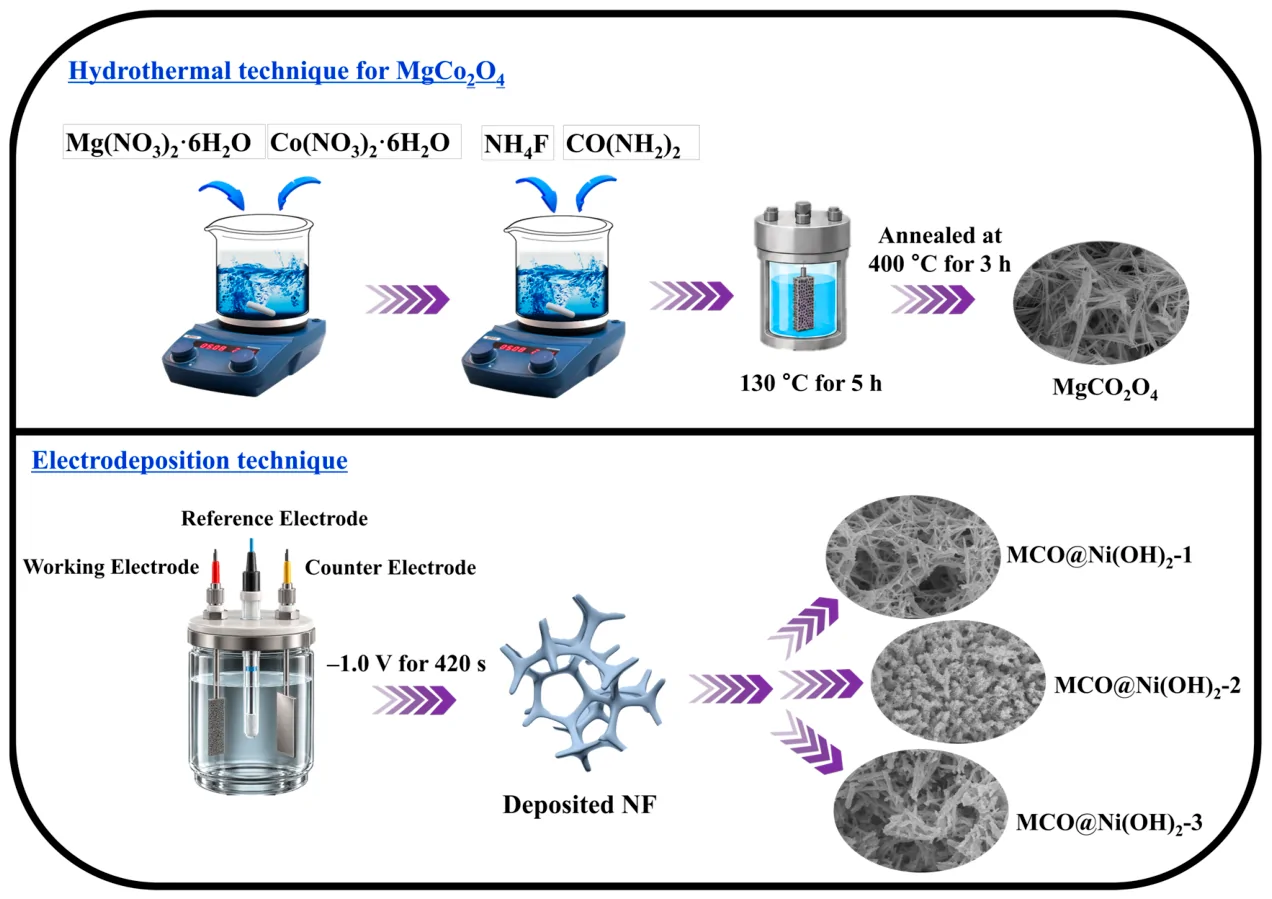
Schematic illustration of synthesis of MCO and MCO@Ni(OH)_2_ nanocomposites.

MgCo_2_O_4_ was grown directly on nickel foam (NF) through a hydrothermal route, as illustrated schematically in Figure 1. Initially, 0.195 g of Mg(NO_3_)_2_·6H_2_O and 0.435 g of Co(NO_3_)_2_·6H_2_O were dissolved in 35 mL of deionized water under magnetic stirring for 10 min to obtain a transparent and uniform precursor solution. Subsequently, 0.035 g of NH_4_F and 0.18 g of CO(NH_2_)_2_, were introduced into the solution, followed by continuous stirring for an additional 1 h to ensure complete dissolution and homogeneous mixing. The resulting precursor solution was transferred into a 50 mL Teflon-lined stainless-steel autoclave. A pre-cleaned piece of nickel foam was positioned vertically inside the liner and fully immersed in the reaction solution. The sealed autoclave was maintained at 130 °C for 5 h and then allowed to cool naturally to room temperature. The as-prepared MgCo_2_O_4_-coated NF was removed, thoroughly rinsed several times with deionized water and ethanol to remove residual precursor species and weakly attached deposits, and then dried at 60 °C for 8–12 h. Finally, the sample was annealed in air at 400 °C for 3 h to improve crystallinity and obtain the MgCo_2_O_4_ electrode, hereafter denoted by MCO.

Ni(OH)_2_ was subsequently electrodeposited onto the MCO-coated nickel foam using a conventional three-electrode configuration. The MCO electrode served as the working electrode, while a platinum plate and Ag/AgCl (3 M KCl) electrode were used as the counter and reference electrodes, respectively. For Ni(OH)_2_ electrodeposition, MCO-coated nickel foam with dimensions of 3 cm × 2 cm was used as the working electrode. Aqueous Ni(NO_3_)_2_·6H_2_O solutions with concentrations of 0.05, 0.10, and 0.15 M were employed as deposition electrolytes. Electrodeposition was carried out potentiostatically at −1.0 V for 420 s. After deposition, the electrodes were removed from the electrochemical cell and rinsed thoroughly several times with deionized water to eliminate residual electrolyte species. The cleaned electrodes were subsequently dried in an oven at 60 °C for 8–12 h. The resulting electrodes were designated as MCO@Ni(OH)_2_-1, MCO@Ni(OH)_2_-2, and MCO@Ni(OH)_2_-3 according to the increasing concentration of the nickel precursor used during electrodeposition. For comparative XPS analysis, a reference Ni(OH)_2_ electrode was prepared on nickel foam under the same electrodeposition conditions used for MCO@Ni(OH)_2_-2. The Ni 2p and O 1s spectra of the reference Ni(OH)_2_ were used solely to evaluate binding-energy changes associated with heterostructure formation.

### 2.3. Material Characterization

The structural, chemical, and morphological characteristics of the prepared electrodes were examined using complementary characterization techniques. The crystalline phases were identified by X-ray diffraction (XRD) using an X’Pert Pro diffractometer (PANalytical, Almelo, The Netherlands) with Cu Kα radiation as the X-ray source. The surface elemental composition and chemical oxidation states were investigated by X-ray photoelectron spectroscopy (XPS) using a Thermo Scientific K-Alpha spectrometer (Cheshire, UK). Surface morphology and microstructural features were analyzed using field-emission scanning electron microscopy (FE-SEM; HITACHI S-4800, Tokyo, Japan). The elemental composition and spatial distribution of the constituent elements were further examined using an energy-dispersive X-ray spectroscopy (EDAX) detector coupled to the SEM system.

### 2.4. Electrochemical Analysis

Electrochemical measurements were conducted using a Biologic WBCS3000 workstation in a standard three-electrode configuration. The catalyst-coated nickel foam was employed as the working electrode, while a Hg/HgO electrode and a platinum plate served as the reference and counter electrodes, respectively. After electrodeposition, the resulting electrodes were cut into 1 cm × 1 cm pieces, corresponding to a geometric working area of 1 cm^2^, for OER measurements. The catalyst mass loadings on the tested electrodes were approximately 4.7, 4.2, 4.6, and 4.4 mg cm^−2^ for MCO, MCO@Ni(OH)_2_-1, MCO@Ni(OH)_2_-2, and MCO@Ni(OH)_2_-3, respectively. All electrochemical tests were performed in nitrogen-saturated 1.0 M KOH electrolyte. The OER activity of the prepared electrodes was evaluated by linear sweep voltammetry (LSV) at a scan rate of 5 mV s^−1^ over a potential range of 0–1.0 V. The measured potentials referenced to Hg/HgO were converted to the reversible hydrogen electrode (RHE) scale according to

E_RHE_ = E_Hg/HgO_ + E°_Hg/HgO_ + 0.0591 × (pH) (1)

where E°_Hg/HgO_ represents the standard potential of the Hg/HgO reference electrode and the pH of 1.0 M KOH was taken as approximately 13.9.

To estimate the electrochemically active surface area, cyclic voltammetry was recorded within a non-Faradaic potential window of 0.1–0.2 V at scan rates of 5, 10, 15, 20, and 25 mV s^−1^. The resulting capacitive current response was used to determine the electrochemical double-layer capacitance (C_dl_), from which the ECSA was estimated. Electrochemical impedance spectroscopy (EIS) was employed to investigate the interfacial charge-transfer characteristics of the electrodes. The measurements were performed using an AC perturbation amplitude of 10 mV over a frequency range from 100 kHz to 0.1 Hz. The durability of the optimized electrocatalyst was further examined by comparing the LSV curves recorded before and after 5000 continuous CV cycles. Long-term operational stability was additionally assessed by chronopotentiometry at a constant current density.

## 3. Result and Discussion

**Figure 2:**
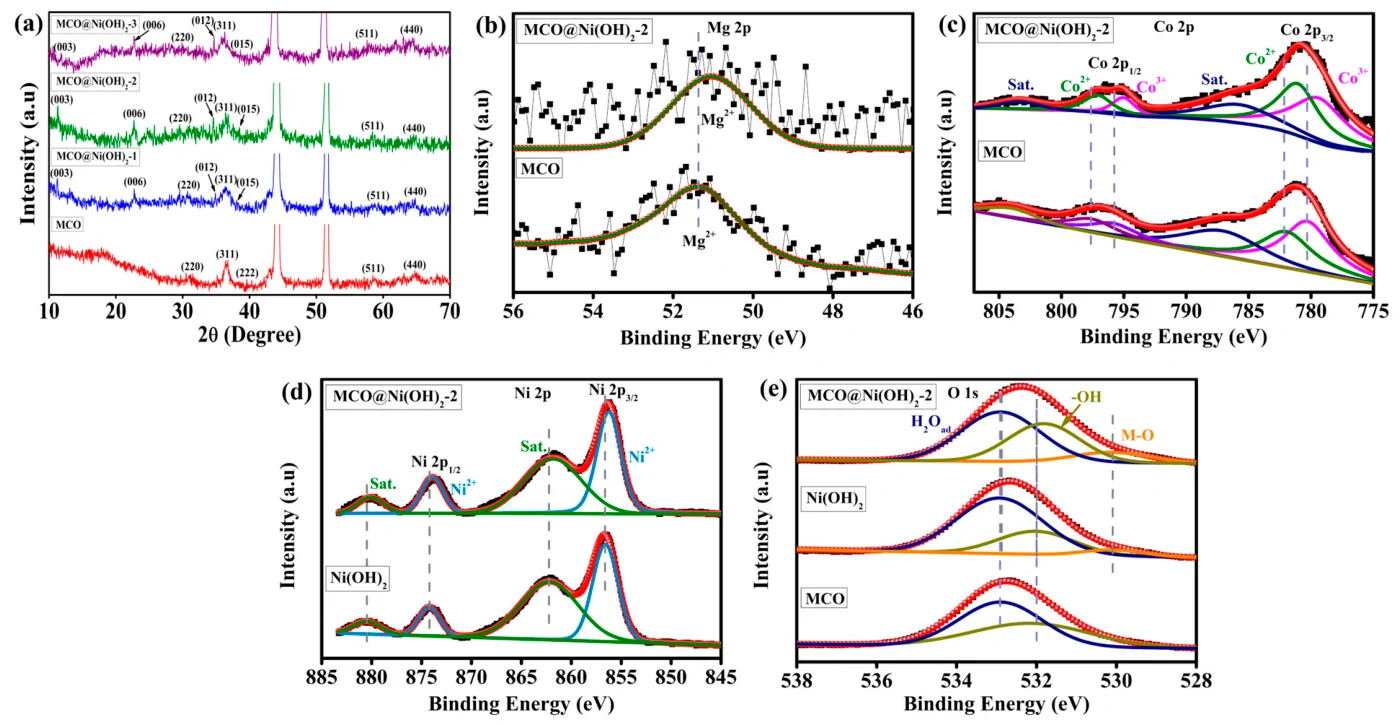
(**a**) XRD spectra of all the composite, high-resolution XPS spectra of (**b**) Mg 2p, (**c**) Co 2p, and (**d**) Ni 2p; and (**e**) O 1s spectra of pristine MCO, Ni(OH)_2_, and MCO@Ni(OH)_2_-2 composite.

To further elucidate the crystalline structure and phase composition of the prepared electrodes, X-ray diffraction (XRD) analysis was performed. Figure 2a shows the diffraction patterns of MCO, MCO@Ni(OH)_2_-1, MCO@Ni(OH)_2_-2, and MCO@Ni(OH)_2_-3. The pristine MCO sample displays characteristic diffraction peaks at approximately 31.4°, 36.7°, 39.5°, 58.4°, and 64.7°, which can be indexed to the (220), (311), (222), (511), and (440) crystallographic planes, respectively. These reflections are consistent with the cubic spinel structure of MgCo_2_O_4_ (JCPDS No. 00-002-1073), confirming the successful formation of the MCO phase [22,23]. Following the electrodeposition of Ni(OH)_2_, additional diffraction reflections appear at approximately 11.3°, 22.6°, 34.4°, and 38.5°. These peaks are assigned to the (003), (006), (012), and (015) planes of rhombohedral Ni(OH) _2_, in agreement with JCPDS No. 00-038-0715 [24,25]. The simultaneous presence of the characteristic reflections of both MgCo_2_O_4_ and Ni(OH)_2_ demonstrates the successful construction of the MCO@Ni(OH)_2_ heterostructure without substantial disruption of the underlying MgCo_2_O_4_ crystalline framework. Moreover, the relative intensity of the Ni(OH)_2_-associated diffraction peaks becomes progressively more pronounced across the MCO@Ni(OH)_2_ series, which is consistent with an increased amount of electrodeposited Ni(OH)_2_ on the MgCo_2_O_4_ surface.

X-ray photoelectron spectroscopy (XPS) was employed to investigate the surface elemental composition and chemical states of the optimized MCO@Ni(OH)_2_-2 electrode. As shown in Figure 2b, the Mg 2p peak of pristine MCO is located at approximately 51.3 eV, whereas that of MCO@Ni(OH)_2_-2 appears at approximately 51.0 eV. These peaks are assigned to Mg^2+^ species coordinated with oxygen within the Mg-O framework of the spinel MgCo_2_O_4_ phase [22,23]. The high-resolution Co 2p spectra in Figure 2c display the characteristic Co 2p_3/2_ and Co 2p_1/2_ spin–orbit components, together with the corresponding satellite features. For pristine MCO, the fitted components located at approximately 780.3 and 795.7 eV are assigned to Co^3+^, while those at approximately 781.9 and 797.4 eV correspond to Co^2+^ species. After Ni(OH)_2_ deposition, the corresponding Co^3+^ contributions shift to approximately 779.59 and 794.60 eV, whereas the Co^2+^ components shift to approximately 781.01 and 796.10 eV in MCO@Ni(OH)_2_-2 [26,27,28]. The simultaneous presence of Co^2+^ and Co^3+^ confirms the mixed-valence nature of cobalt in the MgCo_2_O_4_ spinel structure. As shown in Figure 2d, pristine Ni(OH)_2_ exhibits Ni 2p_3/2_ and Ni 2p_1/2_ peaks at approximately 856.5 and 874.2 eV, respectively. In MCO@Ni(OH)_2_-2, these peaks are located at approximately 856.2 and 874.0 eV, respectively. The corresponding shake-up satellite features further support the presence of Ni^2+^ species associated with Ni(OH)_2_ [24,25]. The detection of these characteristic Ni^2+^ signals provides additional evidence for the successful formation of Ni(OH)_2_ on the MgCo_2_O_4_ surface. The chemical environment of oxygen was further examined from the high-resolution O 1s spectrum shown in Figure 2e. The O 1s envelope can be resolved into several contributions originating from different oxygen species. The higher-binding-energy components at approximately 531.8 and 532.8 eV are associated predominantly with surface hydroxyl species (-OH) and adsorbed oxygen-containing species, including molecular water for pristine MCO, Ni(OH)_2_, and MCO@Ni(OH)_2_-2, respectively, with the slight shift in the MCO@Ni(OH)_2_-2 spectrum [22,27]. The component located at approximately 529.9 eV is attributed to lattice oxygen (O^2-^) associated with metal–oxygen bonds within the crystalline oxide framework [22,29]. Compared with the pristine MCO and Ni(OH)_2_ reference samples, the Mg 2p, Co 2p, and Ni 2p peaks of MCO@Ni(OH)_2_-2 exhibit shifts toward lower binding energies. These negative shifts indicate an increase in local electron density around the corresponding metal centers, suggesting a more electron-rich and relatively reduced chemical environment in the heterostructured electrode [30,31].

**Figure 3:**
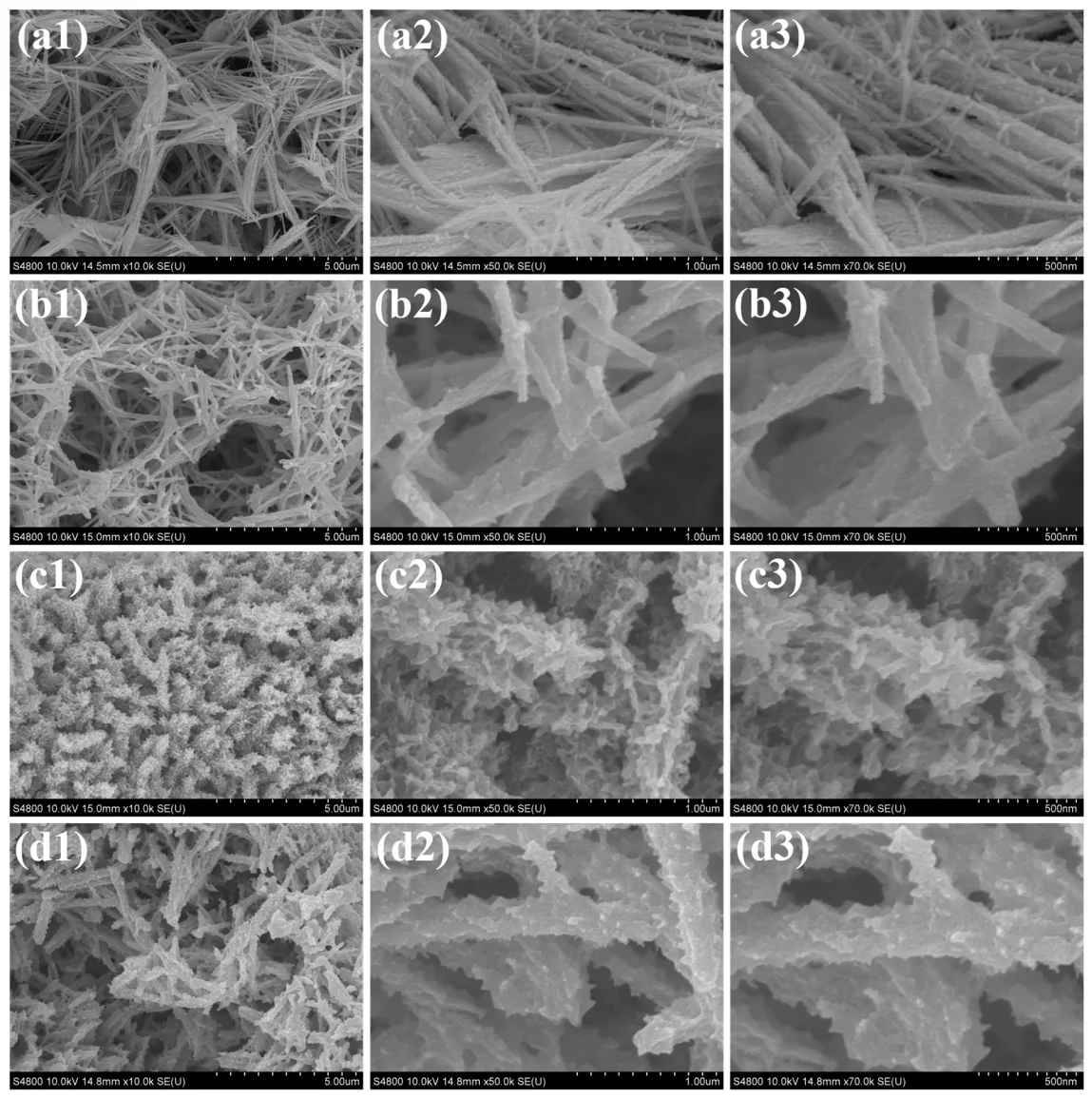
SEM micrograph images of (**a1**–**a3**) MCO, (**b1**–**b3**) MCO@Ni(OH)_2_-1, (**c1**–**c3**) MCO@Ni(OH)_2_-2, and (**d1**–**d3**) MCO@Ni(OH)_2_-3.

The surface morphology and microstructural evolution of the synthesized electrodes were examined by SEM at different magnifications, as shown in Figure 3. The pristine MCO sample in Figure 3(a1–a3) exhibits a well-developed nanorod-like architecture. The elongated nanorods are interwoven with each other, forming an open three-dimensional network. After electrodeposition of Ni(OH)_2_, a clear change in surface morphology is observed. For MCO@Ni(OH)_2_-1 (Figure 3(b1–b3)), thin Ni(OH)_2_ nanosheets begin to grow on the surface of the MgCo_2_O_4_ nanorods, partially covering the underlying rod-like structure. With further deposition, MCO@Ni(OH)_2_-2, as presented in Figure 3(c1–c3), develops a more uniform and well-defined hierarchical MgCo_2_O_4_@Ni(OH)_2_ heterostructure. In this sample, the MCO nanorods are densely covered by interconnected Ni(OH)_2_ nanosheets while maintaining open voids throughout the structure. In contrast, MCO@Ni(OH)_2_-3 (Figure 3(d1–d3)) shows a thicker and more compact Ni(OH)_2_ layer over the MgCo_2_O_4_ nanorods. The excessive growth and partial aggregation may reduce the accessibility of active sites and hinder efficient mass transport, which could account for its lower OER activity compared with MCO@Ni(OH)_2_-2.

**Figure 4:**
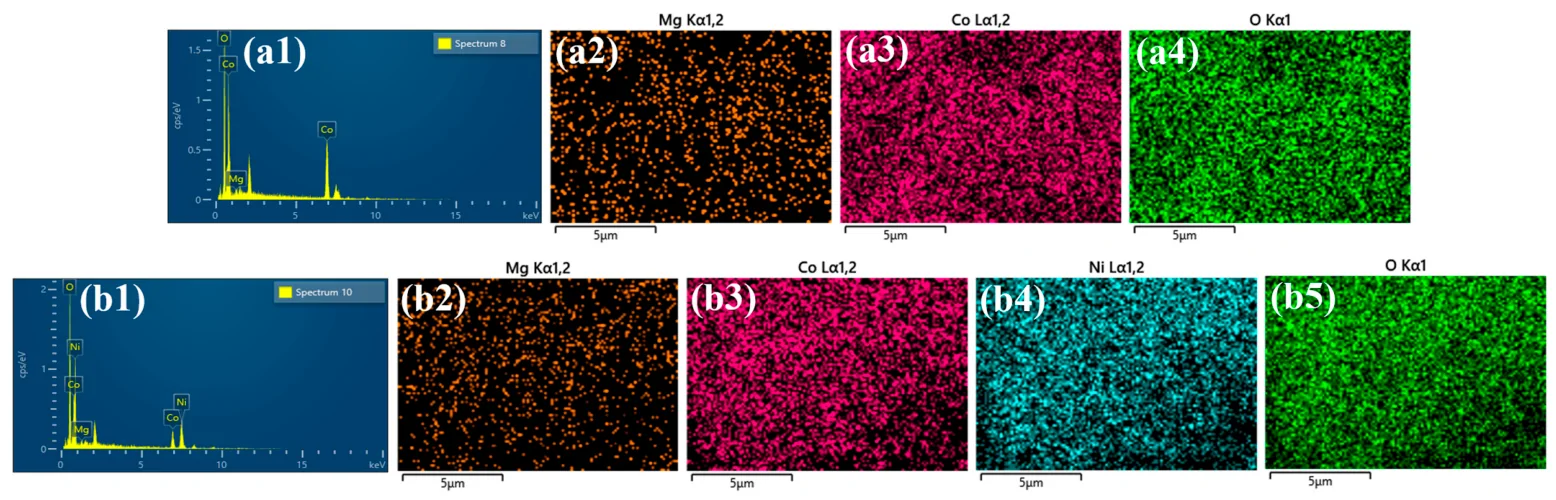
Energy-dispersive X-ray spectroscopy analysis and elemental mapping data of (**a1**–**a4**) MCO and (**b1**–**b5**) MCO@Ni(OH)_2_-2.

The elemental composition and spatial distribution of the constituent elements were further examined by energy-dispersive X-ray spectroscopy and elemental mapping, as shown in Figure 4. The EDX-derived weight percentages are considered semi-quantitative and were not used to determine the exact stoichiometry of MgCo_2_O_4_. The EDAX spectrum of pristine MCO (Figure 4(a1)) confirms the presence of Mg, Co, and O, with corresponding weight percentages of 1.13, 65.65, and 33.23 wt%, respectively. The corresponding elemental mapping data shown in Figure 4(a2–a4) reveal a widespread distribution of Mg, Co, and O throughout the analyzed region, indicating that these elements are well-dispersed within the MCO nanostructure. After electrodeposition of Ni(OH)_2_, the EDAX spectrum of MCO@Ni(OH)_2_-2, demonstrated in Figure 4(b1), shows the additional presence of Ni together with Mg, Co, and O. The measured contents of Mg, Co, Ni, and O are 1.03, 20.52, 42.69, and 35.76 wt%, respectively. The pronounced Ni signal and its relatively high weight fraction provide clear evidence for the successful incorporation of the Ni-containing surface phase. The elemental mapping images of MCO@Ni(OH)_2_-2 provide in Figure 4(b2–b5), further demonstrate that Mg, Co, Ni, and O are distributed across the examined region.

**Figure 5:**
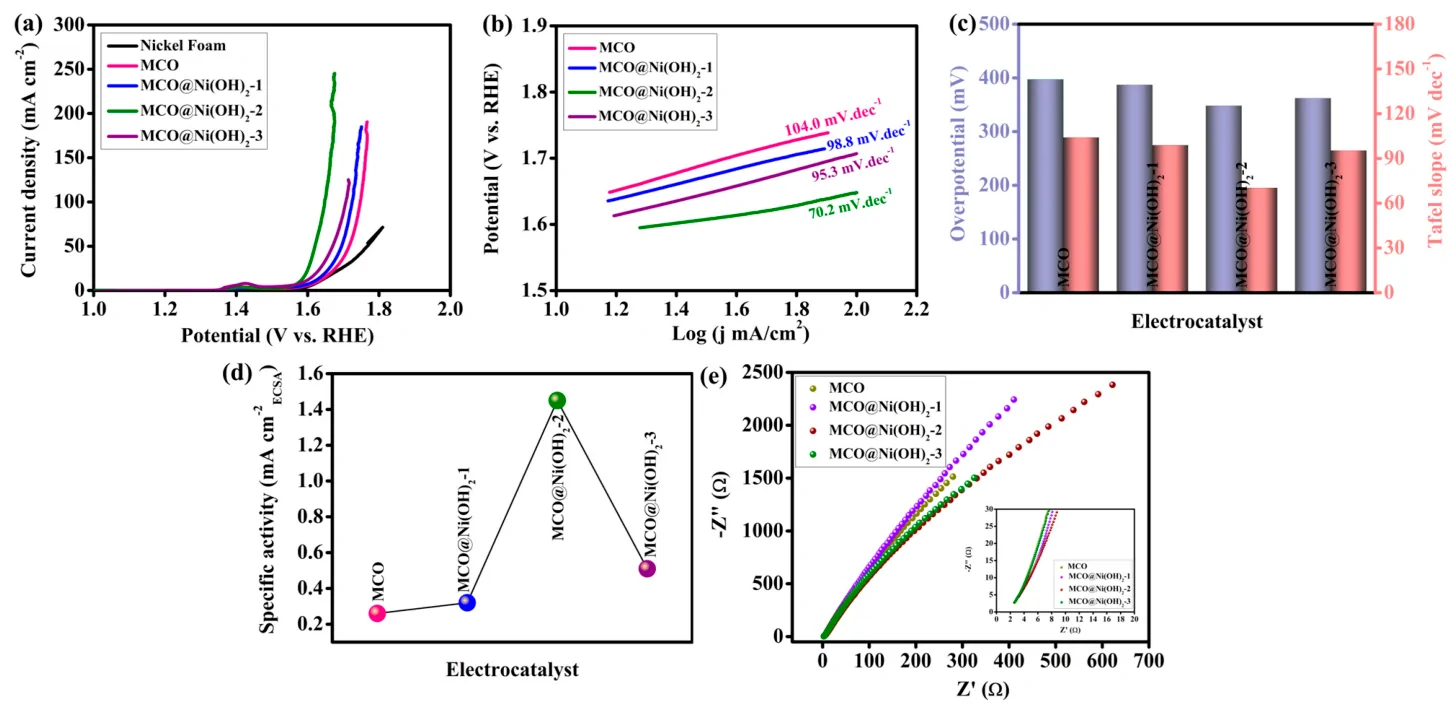
Electrochemical characterizations of electrocatalyst OER performances: (**a**) LSV curves at a 5 mV/s scan rate; (**b**) Tafel slopes; (**c**) valuation of the OER performance concerning overpotential at 10 mA cm^−2^ and Tafel slope; (**d**) ECSA-normalized specific activity; and (**e**) EIS spectra.

The OER performance of the prepared electrodes was evaluated by linear sweep voltammetry (LSV) in alkaline electrolyte, with the polarization curves corrected for uncompensated resistance using manual iR compensation. As shown in Figure 5a, the polarization curves of NF, MCO, MCO@Ni(OH)_2_-1, MCO@Ni(OH)_2_-2, and MCO@Ni(OH)_2_-3 reveal a clear dependence of catalytic performance on the amount of electrodeposited Ni(OH)_2_. Among the investigated electrodes, MCO@Ni(OH)_2_-2 exhibited the most remarkable OER activity, requiring an overpotential of only 348 mV to reach a current density of 10 mA cm^−2^. In comparison, NF, MCO, MCO@Ni(OH)_2_-1, and MCO@Ni(OH)_2_-3 required higher overpotentials of 401, 397, 387, and 362 mV, respectively, as demonstrated in Figure 5c. These results demonstrate that the incorporation of Ni(OH)_2_ substantially enhances the OER activity of MgCo_2_O_4_, with the intermediate Ni(OH)_2_ loading providing the most favorable catalytic response. The decline in performance observed for MCO@Ni(OH)_2_-3 further suggests that excessive Ni(OH)_2_ deposition may partially obstruct accessible active sites or hinder efficient mass and charge transport. Therefore, the improved activity of MCO@Ni(OH)_2_-2 is more reasonably attributed to an optimized balance between the exposed catalytic surface, interfacial contact, and active Ni-containing species, rather than to a simple increase in Ni(OH)_2_ content. To further assess the OER kinetics, Tafel plots were derived from the corresponding polarization curves, as presented in Figure 5b,c. The Tafel slopes of MCO, MCO@Ni(OH)_2_-1, MCO@Ni(OH)_2_-2, and MCO@Ni(OH)_2_-3 were calculated to be 104.0, 98.8, 70.2, and 95.3 mV dec^−1^, respectively. Notably, MCO@Ni(OH)_2_-2 exhibited the smallest Tafel slope of 70.2 mV dec^−1^, indicating more favorable OER kinetics and a faster increase in reaction rate with increasing applied potential. This observation is consistent with its reduced overpotential and confirms the beneficial role of the MgCo_2_O_4_@Ni(OH)_2_ interface in accelerating the multistep oxygen-evolution process. Under anodic polarization, surface Ni(OH)_2_ species can be electrochemically converted into higher-valence NiOOH-type species, which are commonly regarded as catalytically active intermediates during alkaline OER. The comparatively low Tafel slope of 70.2 mV dec^−1^ for MCO@Ni(OH)_2_-2 suggests favorable OER kinetics and may be associated with the electrochemical transformation of Ni-O species into catalytically active NiOOH intermediates [32]. To assess the intrinsic OER activity of the prepared electrodes while reducing the contribution from differences in electrochemically accessible surface area, the measured current densities were normalized by the corresponding ECSA values. The resulting ECSA-normalized specific activities, presented in Figure 5d, were 0.26, 0.32, 1.45, and 0.51 mA cm^−2^_ECSA_ for MCO, MCO@Ni(OH)_2_-1, MCO@Ni(OH)_2_-2, and MCO@Ni(OH)_2_-3, respectively. Among the investigated electrodes, MCO@Ni(OH)_2_-2 delivered the highest specific activity of 1.45 mA cm^−2^_ECSA_, substantially exceeding the values obtained for the other samples. This result demonstrates the superior ECSA-normalized specific activity of MCO@Ni(OH)_2_-2, reflecting its enhanced catalytic performance toward oxygen evolution. Electrochemical impedance spectroscopy (EIS) was further employed to investigate interfacial charge-transfer behavior during the OER, as shown in Figure 5e. The experimental impedance spectra were fitted using an appropriate equivalent-circuit model to extract the charge-transfer resistance (R_ct_). The obtained R_ct_ values were 16.3, 12.7, 2.7, and 8.4 Ω for MCO, MCO@Ni(OH)_2_-1, MCO@Ni(OH)_2_-2, and MCO@Ni(OH)_2_-3, respectively. Among these electrodes, MCO@Ni(OH)_2_-2 displayed the lowest R_ct_ value of 2.7 Ω, indicating the most efficient interfacial electron-transfer process.

**Figure 6:**
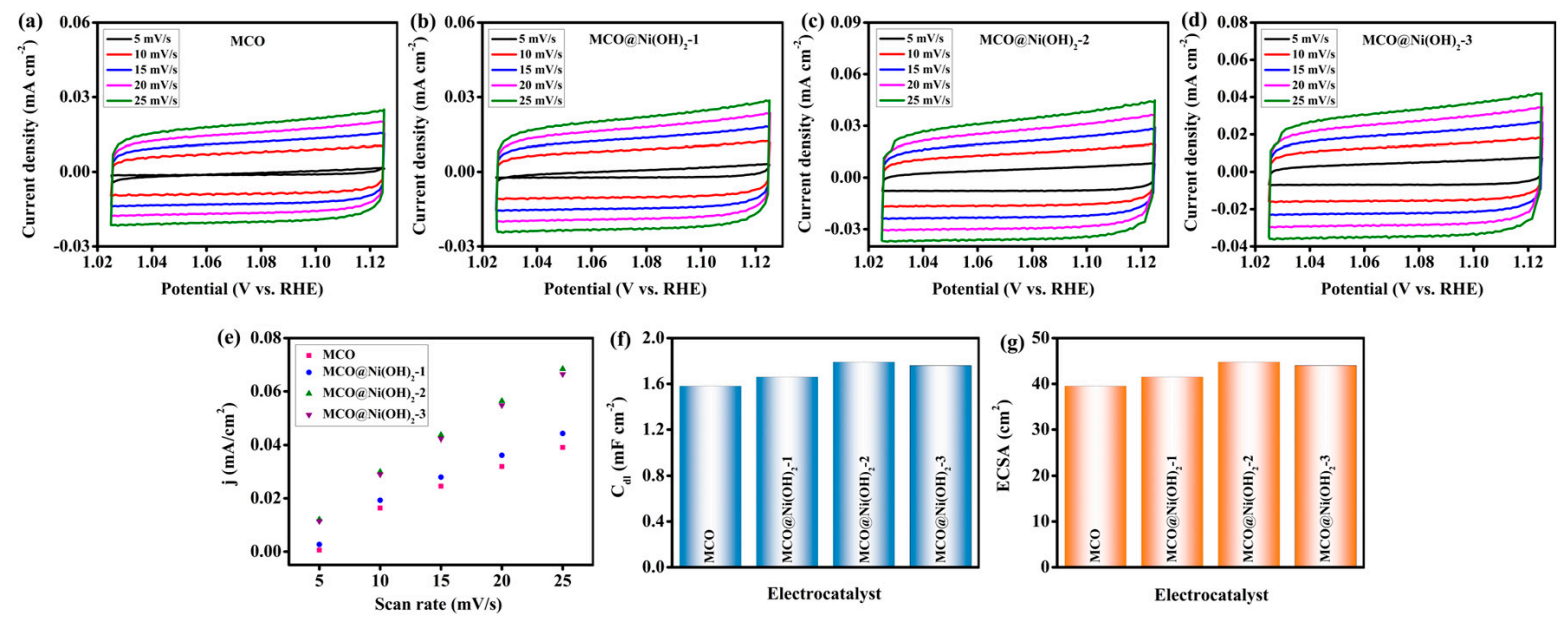
Cyclic voltammetry graph of (**a**) MCO, (**b**) MCO@Ni(OH)_2_-1, (**c**) MCO@Ni(OH)_2_-2, and (**d**) MCO@Ni(OH)_2_-3 at different scan rates. (**e**) 2C_dl_ graph, (**f**) C_dl_ graph, and (**g**) ECSA graph of all of the electrocatalysts.

The cyclic voltammetry (CV) responses of MCO, MCO@Ni(OH)_2_-1, MCO@Ni(OH)_2_-2, and MCO@Ni(OH)_2_-3 were recorded at different scan rates within the non-faradaic potential region, as presented in Figure 6a–d [33]. For all electrodes, the capacitive current density increased progressively with increasing scan rate, reflecting the characteristic charging and discharging behavior of the electrode/electrolyte interface. The approximately linear dependence of the anodic–-cathodic current density difference on scan rate was subsequently used to determine the double-layer capacitance (C_dl_), which provides an indirect indication of the electrochemically accessible surface area. The corresponding linear plots used for C_dl_ determination are shown in Figure 6e. The calculated C_dl_ values (Figure 6f) for MCO, MCO@Ni(OH)_2_-1, MCO@Ni(OH)_2_-2, and MCO@Ni(OH)_2_-3 were 1.58, 1.66, 1.79, and 1.76 mF cm^−2^, respectively. Among the investigated electrodes, MCO@Ni(OH)_2_-2 exhibited the highest C_dl_ value, suggesting a greater number of electrochemically accessible surface sites. This enhancement can be associated with the hierarchical architecture formed by Ni(OH)_2_ nanosheets grown over the MgCo_2_O_4_ nanorod framework, which provides increased interfacial exposure and facilitates electrolyte accessibility. Based on the obtained C_dl_ values, the electrochemically active surface area (ECSA) was estimated, as summarized in Figure 6g. The ECSA values of MCO, MCO@Ni(OH)_2_-1, MCO@Ni(OH)_2_-2, and MCO@Ni(OH)_2_-3 were calculated to be 39.5, 41.5, 44.75, and 44.0 cm^2^, respectively. The highest ECSA obtained for MCO@Ni(OH)_2_-2 is consistent with its superior OER performance, indicating that the optimized Ni(OH)_2_ deposition increases the number of accessible catalytic sites. Although MCO@Ni(OH)_2_-2 and MCO@Ni(OH)_2_-3 possess comparable ECSA values, their intrinsic electrochemical behavior is different. The superior activity of MCO@Ni(OH)_2_-2 is associated with its optimized Ni(OH)_2_ nanosheet coverage, lower charge-transfer resistance, and faster OER kinetics. In contrast, excessive Ni(OH)_2_ deposition in MCO@Ni(OH)_2_-3 produces a thicker and more compact surface layer, which can hinder charge transfer and product transport despite maintaining a similar nominal ECSA.

**Figure 7:**
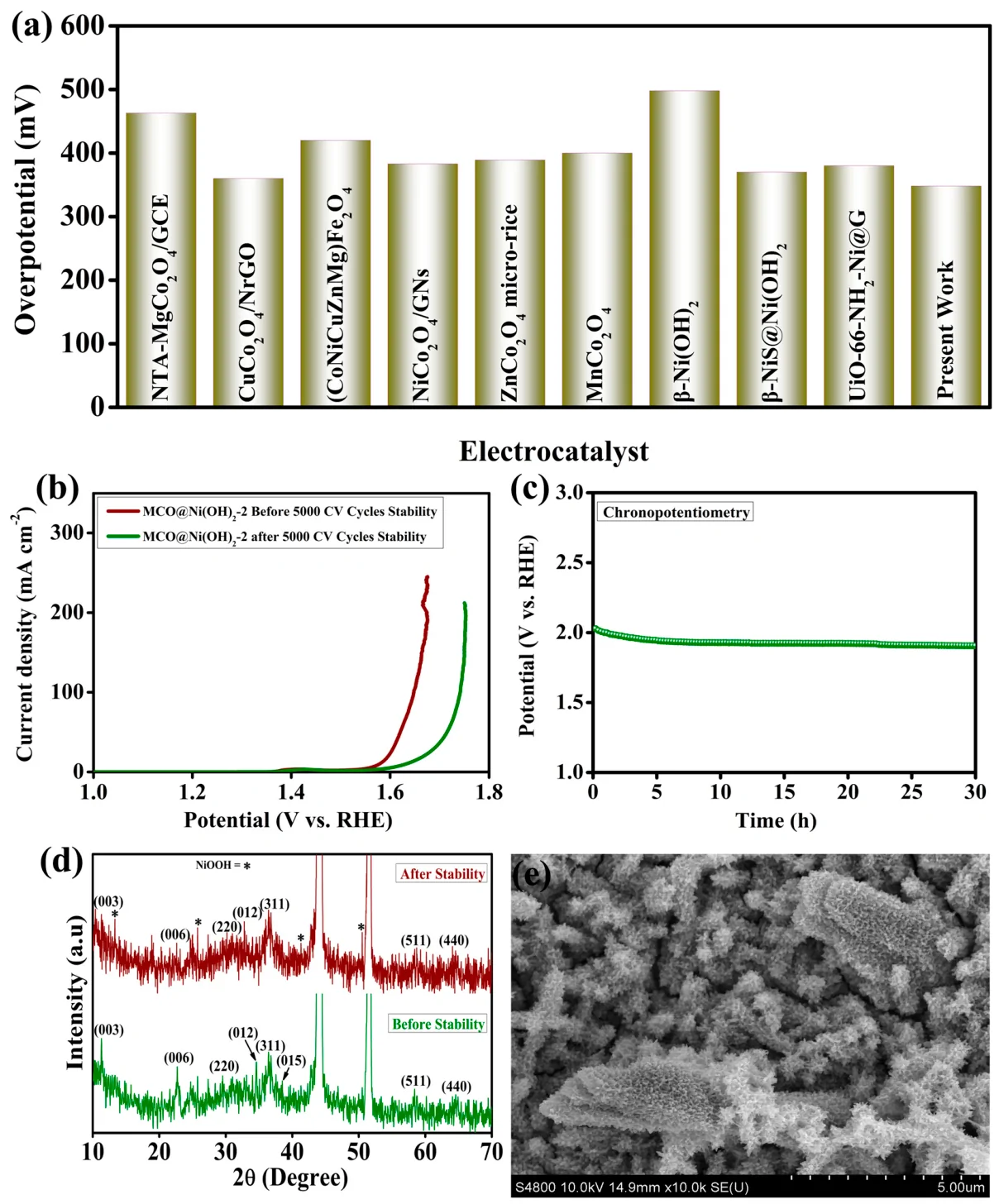
(**a**) Comparative graph of overpotential with recently reported work; (**b**) LSV curves of MCO@Ni(OH)_2_-2 before and after 5000 CV cycles; (**c**) chronopotentiometry analysis; and after-stability (**d**) XRD and (**e**) SEM of MCO@Ni(OH)_2_-2.

The OER performance of the optimized MCO@Ni(OH)_2_-2 electrode was compared with previously reported electrocatalysts, as summarized in Figure 7a and Table 1. In addition to catalytic activity, long-term operational durability is an essential criterion for evaluating the practical applicability of an OER electrocatalyst. Therefore, the electrochemical stability of MCO@Ni(OH)_2_-2 was investigated in 1.0 M KOH through accelerated CV cycling and chronopotentiometric measurements. For the accelerated durability test, the electrode was subjected to 5000 continuous CV cycles at a scan rate of 50 mV s^−1^. The polarization curves recorded before and after cycling were compared to evaluate the retention of OER activity as shown in Figure 7b. After 5000 cycles, MCO@Ni(OH)_2_-2 needed an overpotential of about 401 mV to reach 10 mA cm^−2^, compared with the initial value of 348 mV. This increase in overpotential shows that the catalyst partially maintained its OER activity, although some loss in performance was observed after long-term cycling. The long-term operational stability of MCO@Ni(OH)_2_-2 was further examined by chronopotentiometry at a constant current density of 10 mA cm^−2^ for 30 h, as shown in Figure 7c. The electrode maintained a relatively stable potential response throughout the measurement period, demonstrating its ability to sustain continuous OER operation under alkaline conditions. The small variation in potential observed during prolonged electrolysis may arise from gradual changes at the catalyst/electrolyte interface, accumulation and release of oxygen bubbles, and possible surface reconstruction under anodic polarization [34,35]. To investigate the structural changes induced by prolonged OER operation, the optimized electrode was examined by XRD after the stability test, as shown in Figure 7d. The major diffraction features corresponding to the original MCO@Ni(OH)_2_-2 electrode remain detectable after cycling, indicating that the bulk crystalline framework is at least partly retained. However, several reflections associated with the pristine material become noticeably weaker or are no longer clearly observed, suggesting that electrochemical operation induces structural modification within the surface or near-surface region. In addition, new diffraction peaks appear at approximately 12.9°, 25.7°, 41.3°, and 50.5°, which can be indexed to the (003), (006), (104), and (108) planes of NiOOH, respectively, in agreement with JCPDS No. 00-006-0075 [36]. The attenuation of the parent-phase reflections, together with the appearance of NiOOH-related peaks, suggests partial transformation of the Ni-containing surface species into an oxyhydroxide-rich phase during OER. The morphological condition of the electrode after prolonged electrocatalysis was further evaluated by post-stability SEM analysis, as presented in Figure 7e. The electrode largely retains its interconnected nanostructured architecture after the stability test, indicating that the overall framework remains mechanically intact under anodic operating conditions. Nevertheless, localized surface accumulation and partial morphological rearrangement are observed compared with the pristine electrode. These changes suggest that the catalyst undergoes a certain degree of surface reconstruction during extended OER operation while preserving the main structural framework.

## 4. Conclusions

In summary, a hierarchical MgCo_2_O_4_@Ni(OH)_2_ heterostructure was successfully fabricated by combining hydrothermal growth of MgCo_2_O_4_ nanorods with subsequent electrodeposition of Ni(OH)_2_ nanosheets. Among the prepared electrodes, MCO@Ni(OH)_2_-2 exhibited the best OER performance, requiring an overpotential of 348 mV at 10 mA cm^−2^, compared with 401, 397, 387, and 362 mV for NF, MCO, MCO@Ni(OH)_2_-1, and MCO@Ni(OH)_2_-3, respectively. The optimized electrode also displayed the lowest Tafel slope of 70.2 mV dec^−1^ and the smallest charge-transfer resistance of 2.7 Ω, indicating improved OER kinetics and more efficient interfacial charge transport. After 5000 CV cycles, the electrode required an reasonable overpotential at 10 mA cm^−2^, indicating retention of substantial catalytic activity despite some performance loss during prolonged cycling. The enhanced performance of MCO@Ni(OH)_2_-2 can therefore be associated with the optimized Ni(OH)_2_ nanosheet coverage, increased accessible surface area, and effective interfacial coupling between the MgCo_2_O_4_ nanorod and Ni(OH)_2_. These findings demonstrate the potential of interface-engineered oxide/hydroxide architectures for developing efficient and earth-abundant electrocatalysts for alkaline oxygen evolution.

## Figures and Tables

**Table 1:** OER performance of recently reported work.

Electrocatalyst	Substrate	Electrolyte	Overpotential (mV@10 mA cm^−2^)	Ref.
NTA-MgCo_2_O_4_/GCE	GCE	0.1 M KOH	463	[37]
CuCo_2_O_4_/NrGO	GCE	1 M KOH	360	[38]
(CoNiCuZnMg)Fe_2_O_4_	-	1 M KOH	420	[39]
NiCo_2_O_4_/GNs	Graphene nanosheets	1 M KOH	383	[40]
ZnCo_2_O_4_ micro-rice	GCE	1 M KOH	389	[41]
MnCo_2_O_4_	carbon cloth	1 M KOH	400	[42]
β-Ni(OH)_2_	GCE	1 M KOH	498	[43]
β-NiS@Ni(OH)_2_	Ni Foam	1 M KOH	370	[44]
UiO-66-NH_2_-Ni@G	GCE	1 M KOH	380	[45]
MCO@Ni(OH)_2_-2	Ni Foam	1 M KOH	348	Present Work

## Data Availability

The original contributions presented in this study are included in the article. Further inquiries can be directed to the corresponding author.
